# Cryo-EM structure of lysenin pore elucidates membrane insertion by an aerolysin family protein

**DOI:** 10.1038/ncomms11293

**Published:** 2016-04-06

**Authors:** Monika Bokori-Brown, Thomas G. Martin, Claire E. Naylor, Ajit K. Basak, Richard W. Titball, Christos G. Savva

**Affiliations:** 1 Biosciences, College of Life and Environmental Sciences, University of Exeter, Stocker Road, Exeter EX4 4QD, UK; 2 MRC Laboratory of Molecular Biology, Francis Crick Avenue, Cambridge Biomedical Campus, Cambridge CB2 0QH, UK; 3 Department of Biological Sciences, Birkbeck College, Malet Street, London WC1E 7HX, UK

## Abstract

Lysenin from the coelomic fluid of the earthworm *Eisenia fetida* belongs to the aerolysin family of small β-pore-forming toxins (β-PFTs), some members of which are pathogenic to humans and animals. Despite efforts, a high-resolution structure of a channel for this family of proteins has been elusive and therefore the mechanism of activation and membrane insertion remains unclear. Here we determine the pore structure of lysenin by single particle cryo-EM, to 3.1 Å resolution. The nonameric assembly reveals a long β-barrel channel spanning the length of the complex that, unexpectedly, includes the two pre-insertion strands flanking the hypothetical membrane-insertion loop. Examination of other members of the aerolysin family reveals high structural preservation in this region, indicating that the membrane-insertion pathway in this family is conserved. For some toxins, proteolytic activation and pro-peptide removal will facilitate unfolding of the pre-insertion strands, allowing them to form the β-barrel of the channel.

## Abstract (web summary)

Lysenin is member of the aerolysin family of small ß-barrel pore-forming toxins that include virulence factors from several human and animal pathogens. Here the authors determine the structure of the lysenin pore by single particle cryo- EM and propose a conserved pore formation mechanism for the aerolysin protein family.

## Graphical abstract

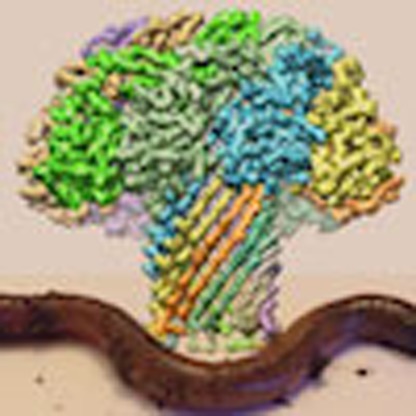

Lysenin, the defence protein of the earthworm *Eisenia fetida*, is produced by coelomocytes in the body fluid that form part of the earthworm's immune system^1^. It is a member of the aerolysin family of small β-pore-forming toxins (β-PFTs) that include key virulence factors of a large number of bacterial pathogens, such as aerolysin produced by *Aeromonas* spp^2^, *Clostridium perfringens* epsilon toxin^3^ and *Clostridium septicum* α-toxin^4^, while *Bacillus thuringiensis* parasporin-2 has cytocidal activity against human cancer cells^5^. Despite their low sequence homology, the structures of their water-soluble monomeric forms reveal that they all share a structurally conserved domain, termed the pore-forming module (PFM), which is known to contribute to the β-barrel pore^6^.

Like most PFTs, lysenin is secreted as an inactive, water-soluble monomer^7^. On binding to sphingomyelin (SM) in the eukaryotic cell membrane, the toxin undergoes significant changes to its secondary structure^8^ and forms oligomeric pre-pores on the membrane surface, as identified by electron crystallographic^9^ and high-speed atomic force microscopy studies^10^,^11^, before inserting into the membrane, which leads to pore formation and consequent cell lysis^12^,^13^.

The crystal structure of monomeric lysenin revealed that the toxin is organized into two main structural domains: (1) the elongated PFM at the N terminus that contains a SM-binding site, as well as the membrane-insertion loop, long thought to be the region that reorganizes into a β-hairpin during membrane insertion, and (2) the C-terminal beta-trefoil domain that has been crystallized bound to phosphocholine (POC)^9^. Investigation of the structure of monomeric lysenin bound to SM revealed that both the POC headgroup and one acyl chain tail of SM are required for toxin binding^9^.

Although several structures have been determined in the past for the monomeric forms of toxins from the aerolysin family^2^,^3^,^5^,^9^,^14^,^15^,^16^,^17^,^18^, there is no reported atomic structure for their active, pore forms. Here we report the structure of lysenin in its nonameric membrane-inserted form determined by single particle cryo-EM, to 3.1 Å resolution. The structure provides the first atomic resolution view into the membrane-inserted state of a member of the aerolysin family and gives insights into how other members of this family may be activated and assemble into their respective pore forms.

## Results

### Structure determination of the lysenin pore

**Figure 1:**
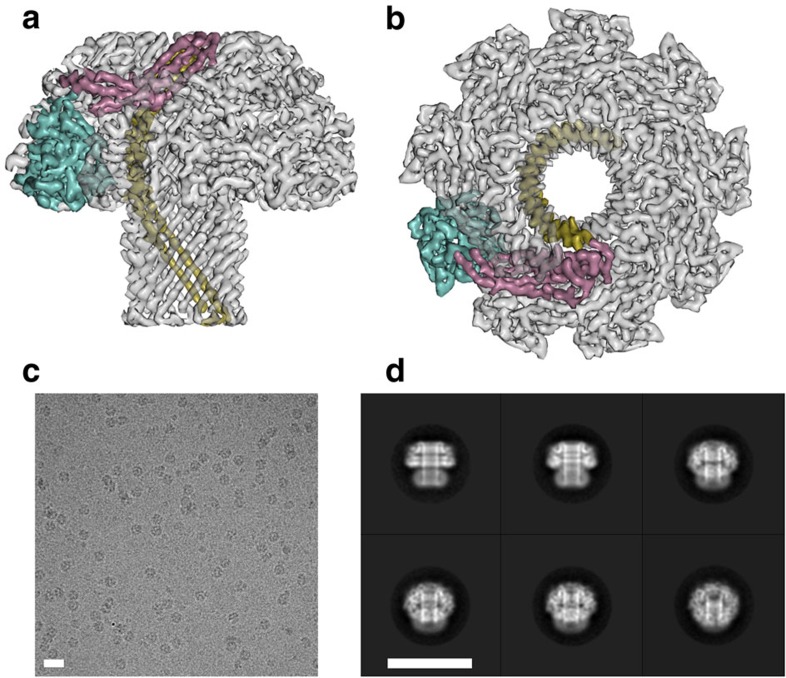
Cryo-EM reconstruction of the lysenin pore. Surface representation of the sharpened 3.1 Å map of lysenin shown from the side (**a**) and extracellular views (**b**). Side chain densities are clearly visible on the β-barrel pore. The three domains of a lysenin subunit are indicated: β-hairpin domain (olive), cap domain (pink) and receptor-binding domain (cyan). Representative cryo-electron micrograph of lysenin complexes on graphene oxide (**c**) taken at −2.3 μm defocus on an FEI Titan Krios with a Gatan K2 Summit detector. Characteristic 2D class averages of the lysenin oligomer (**d**). Scale bars, 20 nm.

Lysenin monomers oligomerize and form SDS-resistant channels on interaction with lipid bilayers containing SM^19^,^20^. We reasoned that we could assemble lysenin oligomers that are biologically active (Supplementary Fig. 1a) in liposomes and then solubilize these complexes in detergent and treat them as single particles for cryo-EM analysis. Solubility screening using ultracentrifugation identified dodecylmaltoside as a suitable detergent (Supplementary Fig. 1b). Because of the tendency of the sample to aggregate at high concentrations (Supplementary Fig. 2a), we imaged particles enriched against a continuous support film. Due to the relatively small size of the expected lysenin complex (210 kDa for a hexamer and 315 kDa for a nonamer, based on the molecular weight of 35 kDa for the His-tagged monomer), we deposited samples on TEM holey-carbon grids that were overlaid with graphene oxide^21^ (Fig. 1c, Supplementary Figs 2b,c and 3). Compared with amorphous carbon, the near-electron transparent nature of graphene oxide yielded higher signal to noise ratios and improved alignment accuracies. Image analysis and two-dimensional (2D) classification of 53,779 particles led to a data set of 42,830 particles, which were used for an initial 3D reconstruction at 3.4 Å resolution with C9 symmetry or 4.2 Å with no symmetry imposed. Movie frame processing for beam-induced particle motion tracking and B-factor-weighted radiation-damage correction^22^ followed by 3D classification resulted in a subset of ‘shiny' particles used for further refinement. This produced a final symmetrized map at 3.1 Å resolution (Fig. 1a,b and Supplementary Fig. 4), approaching the Nyquist limit of 2.9 Å for the pixel size used. The local resolution in the map varies only very little from the overall estimate, with minor parts at the tip of the complex resolved at 4 Å (Supplementary Fig. 4c). The map allowed unambiguous placement of the Cα main-chain and almost all side chains. No other oligomeric species were identified during the 2D (Fig. 1d) and 3D classification steps, indicating that lysenin pores are almost exclusively nonameric. This symmetry is consistent with previous cross-linking studies^20^ and is the second identified example of a protein from the aerolysin family, alongside monalysin, to form nonameric assemblies^17^.

### Architecture of the lysenin assembly

**Figure 2:**
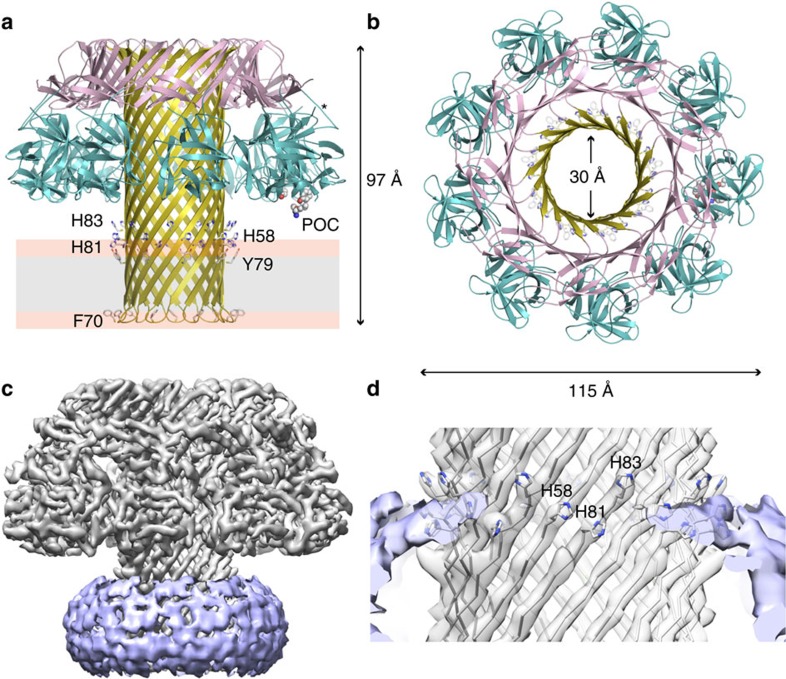
Atomic model of the lysenin pore. Cartoon representation of lysenin shown from the side (**a**) and extracellular views (**b**). The three domains of lysenin are coloured as in Fig. 1: β-hairpin domain (olive), cap domain (pink) and receptor-binding domain (cyan). The dimensions of the complex and the pore are shown as Cα–Cα measurements and the approximate location of the bilayer is drawn with the phospholipid head groups shown in orange and the hydrophobic core in grey. The aromatic rings of Phe70 and Tyr79 are shown in stick representation, as are the histidine residues directly above the Tyr79 ring. The phosphocholine-binding site residues (POC) are shown as spheres and the asterisk indicates the flexible coil connecting the receptor-binding domain to the cap domain. (**c**) Surface representation of the Gaussian filtered detergent shell (purple) around the sharpened lysenin map. (**d**) Close view of the extracellular side of the detergent belt highlighting the three histidine residues.

The lysenin pore resembles the mushroom-shaped pore complexes of the α-haemolysin family of small β-PFTs^23^,^24^ (Fig. 2a,b and Supplementary Fig. 5), although structures of their water-soluble monomers are fundamentally different^9^,^25^,^26^. Monomeric lysenin has previously been divided into two domains: the N-terminal PFM and the C-terminal beta-trefoil domain^9^. However, because of the large structural rearrangement associated with its membrane insertion, we have changed the domain nomenclature. Thus, each of the nine monomers in the membrane-inserted state can be divided into three domains: the N-terminal cap domain (Glu10–Ser33 and Pro108–Ile156), the β-hairpin domain (Val34–Ile107) and the C-terminal receptor-binding domain (Val157–Gly297) (Fig. 2a,b). Neighbouring interactions between the subunits bury ∼3,300 Å^2^ and involve 90 residues, which are distributed among the three domains and include hydrogen bonds and several salt bridges. A random coil from Val157 to Glu167 connects the cap domain to the receptor-binding domain (Fig. 2a, asterisk). This region is flexible^9^ and plays an important role in the transition to the membrane-inserted state. The C-terminal receptor-binding domain has remained mostly unchanged relative to the soluble monomer crystal structure, with an r.m.s.d. (Cα–Cα) of 0.6 Å, and consists of 12 β-sheets and a short 3_10_ helix arranged in a β-trefoil motif.

### Structure of the β-barrel pore

In contrast to the α-haemolysin family where the β-barrel begins close to the membrane-spanning region (Supplementary Fig. 5), the long β-barrel in lysenin spans the length of the assembly. The membrane-spanning region, measuring ∼35 Å, is clearly visible in filtered maps as a detergent-bound shell that delineates the boundaries of the lipid bilayer (Fig. 2c) and is lined on the outside by hydrophobic residues, such as Phe, Tyr, Val and Ile. In the water-soluble lysenin monomer, these residues reside within the hydrophobic core of the PFM and are thus shielded from solvent. Two aromatic rings, one formed by Phe70 and the other by Tyr79 lie on the intracellular and extracellular sides of the transmembrane region, respectively (Fig. 2a). These rings are common to β-barrel membrane proteins^23^,^24^,^27^ and are typically found snorkelling at the interface between the polar lipid head groups and the hydrophobic acyl chains helping to stabilize the orientation of the β-barrel^28^. In addition to the aromatic rings, lysenin also contains a histidine triad just above the Tyr79 ring (histidine residues 58, 81 and 83) (Fig. 2a,d) that is situated within the edge of the detergent belt, suggesting these residues may interact with the extracellular leaflet of the bilayer. The β-turn of each hairpin ends in Glu71, which protrudes towards the intracellular side of the bilayer. The lumen of the β-barrel is lined with charged and polar residues, including 12 threonine and 9 serine residues. An electrostatic potential map of the lumen surface (Supplementary Fig. 6) reveals four regions of negative charge, including one on either entrance to the β-barrel and two in the middle of the pore. The charge distribution is consistent with planar-bilayer channel measurements that suggest a cation-selective pore^20^.

### Interaction with lipids

Monomeric lysenin has previously been crystallized bound to SM and POC ligands^9^, although these structures were clearly of the water-soluble state. POC was crystallized bound to the C-terminal β-trefoil domain of lysenin in a pocket lined by Lys185, Ser227, Gln229, Tyr233 and Tyr282 (ref. 9). In the pore form of lysenin this pocket is ideally situated for interaction with the phosphatidylcholine or SM head groups as it lies on the tip of the receptor-binding domain directly above the target cell membrane (Fig. 2a, ‘POC'). SM was identified bound to the N-terminal PFM, and the residues that interacted with SM in the monomer structure (Lys21, Tyr24, Tyr26, Gln117 and Glu128) are buried within the cap domain of the lysenin pore (Supplementary Fig. 4d). Moreover, we did not find additional densities corresponding to lipids in the C9 symmetrized map or in the 4.2 Å asymmetric reconstruction of lysenin in this location. It is possible that SM recognition at this site occurs prior to oligomerization and membrane insertion as proposed previously^9^. It is also feasible that the high detergent concentrations used for efficient pore extraction from liposomes may have dissociated any bound lipid molecules from an alternative SM-binding site in the lysenin pore.

### Transition to the membrane-inserted state

**Figure 3:**
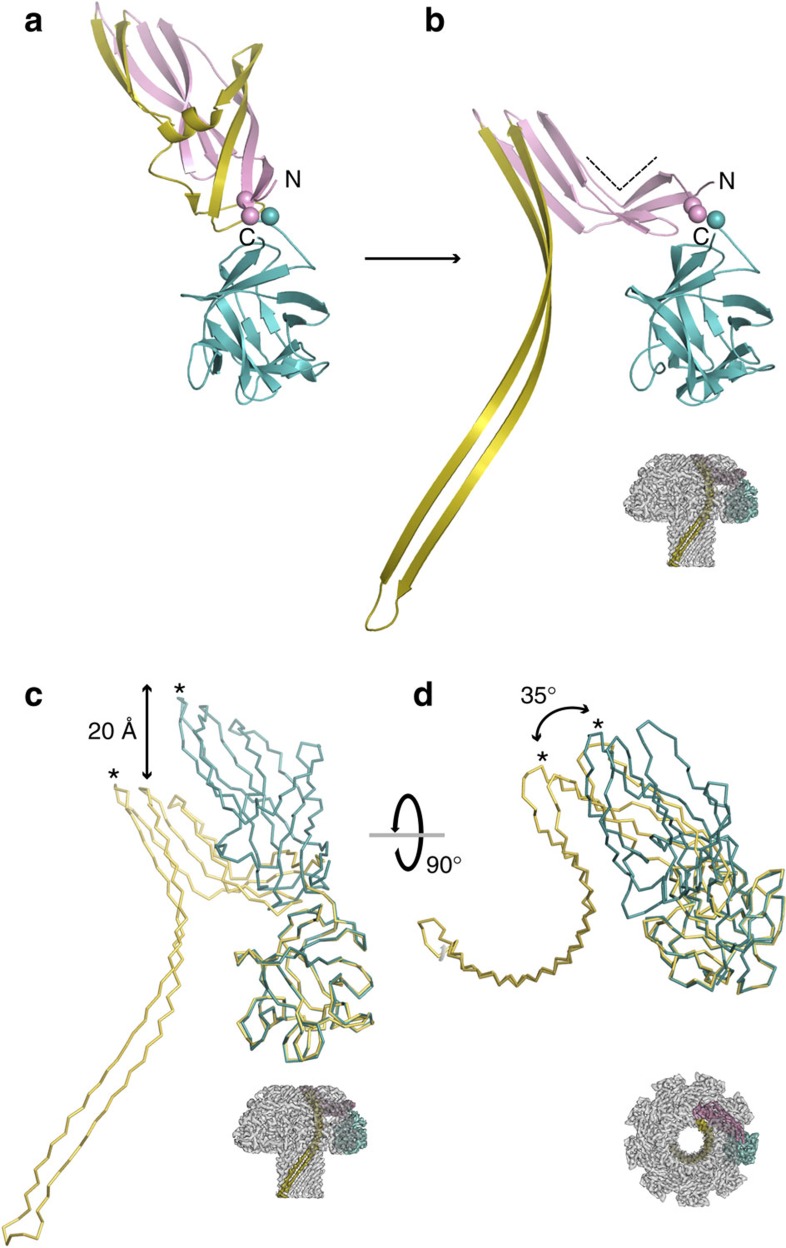
Transition of water-soluble lysenin monomer to its membrane-inserted state. (**a**) Cartoon representation of the lysenin monomer (PDB ID 3ZXD) and its membrane-inserted state (**b**) with both molecules aligned in respect to the receptor-binding domain in the orientation indicated (inset). The dashed lines in **b** indicate the bend in the cap domain and the hinge residues (Val157–Arg159) are shown as spheres. The colouring scheme is as shown in Fig. 2a, with the N- and C-termini indicated. Ribbon representation of the superimposed water-soluble monomer (cyan) and its membrane-inserted form (olive) in reference to the receptor-binding domain, as seen from the side (**c**) and extracellular views (**d**). The asterisks point to Ser31 in both structures. The 20 Å drop in height and the 35° rotation towards the lumen of the cap domain are indicated.

Figure 3 and Supplementary Movie 1 depict the structural rearrangement between the soluble monomer structure (Fig. 3a) and its membrane-inserted, oligomeric state (Fig. 3b). Hinging on the flexible coil between Val157 and Arg159 (Fig. 3a,b, spheres), the N-terminal cap domain swivels downwards by ∼20 Å (Fig. 3c) and rotates inwards towards the central lumen by ∼35° (Fig. 3d). The vertical collapse is consistent with AFM observation of lysenin on planar bilayers, which found a height reduction of up to 3 nm on membrane insertion^10^. This movement induces a bend in the middle of the relatively flat cap domain of the soluble monomer (Fig. 3b, dashed lines) and results in each cap domain being positioned on the top of the adjacent subunit's receptor-binding domain (Supplementary Movie 1). Extension of the long 74 residue β-hairpin requires the complete rearrangement of 5 β-strands and a 3_10_ helix from the PFM of monomeric lysenin^9^ into 2 new β-strands, as highlighted in Fig. 3a,b. The β-hairpin begins near the extracellular side of the pore lumen and curves towards the intracellular side by almost 270° (Fig. 3d). Thus, the β-barrel pore of lysenin is made up of 3 times as much polypeptide as was previously thought (24 residues)^9^.

## Discussion

The cryo-EM structure of the 315 kDa lysenin pore at 3.1 Å resolution represents the first atomic resolution pore structure for a member of the aerolysin family of small β-PFTs, unveiling the transition of the water-soluble monomer into its membrane-inserted, oligomeric state. The structure sets the framework for future studies on how other members of this family may achieve pore formation and opens the pathway for the design of new therapeutics aimed at the functional disruption of pore formation for this family.

**Figure 4:**
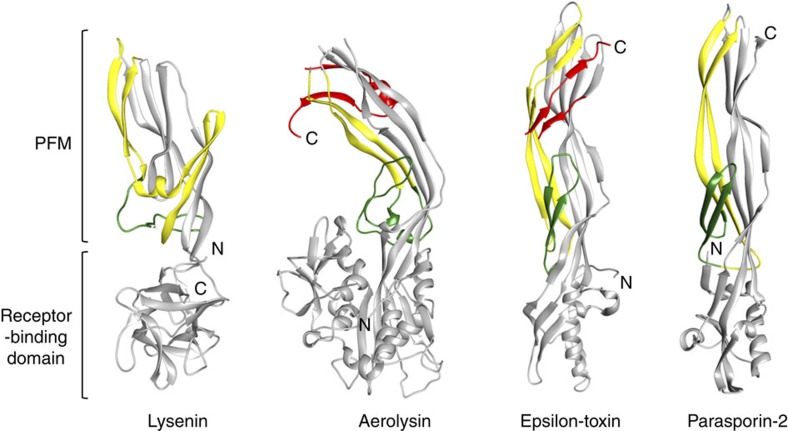
Conserved mechanism of membrane insertion by the aerolysin family. Comparison of the water-soluble monomeric structure of lysenin with three other members of the aerolysin family. The putative β-barrel-contributing regions in aerolysin (1PRE), epsilon toxin (3ZJX) and parasporin-2 (2ZTB) are coloured in green for the previously thought membrane-insertion loops and yellow for the flanking pre-insertion strands identified in this study. Collectively, these regions form the β-barrel of the lysenin pore. The two pre-insertion strands are typically located on the edge of the toxins and isolated from the main body (grey). In the case of aerolysin and epsilon toxin, the pre-insertion strands participate in β-sheets with the CTPs (red), thus stabilizing the soluble monomeric state. In parasporin-2, the N-terminal peptide, which was not present in the crystal structure of the activated toxin, has the greatest effect on toxin activity, and was hypothesized to interact with the insertion loop, thus acting as a safety-lock^5^.

Members of the aerolysin family share an overall basic domain arrangement as evident by their crystal structures^2^,^3^,^5^,^9^ (Fig. 4). They typically contain one or more receptor-binding domains and a PFM. The latter domain contains a flexible insertion loop, long thought to be the region that reorganizes into a β-hairpin during membrane insertion, due to its amphipathic character of alternating hydrophilic/hydrophobic residues^29^ (Fig. 4, coloured in green). The pore structure of lysenin reveals that the β-barrel recruits polypeptide from both the amphipathic loop and the two β-strands that feed into it (Fig. 3b), from here onwards referred to as the pre-insertion strands. Closer examination of all solved monomer structures from the aerolysin family reveals that the hypothetical insertion loops in these proteins are also flanked by predominantly β-strand polypeptides that lead up to the top of the PFMs of each protein (some examples are shown in Fig. 4, yellow strands). Interestingly, in all cases, these pre-insertion strands are positioned on the edge of the toxins, isolated from the main body of the protein, making limited contact in the form of short β-sheets. It is also notable that the combined lengths of the pre-insertion strands and insertion loops in all these proteins do not vary significantly (mean of 75 residues and a s.d. of 8 residues) and are rich in serine and threonine residues (Thr+Ser=23–44%)^5^,^30^, most of which are found lining the lumen of the lysenin β-barrel pore. Due to the structural homology shared within the PFMs of the aerolysin family, we expect that other members may follow similar strategies for monomer reorganization, which will involve the unfolding of the pre-insertion strands for β-barrel formation and the remainder of the PFM forming the caps of their respective mushroom-shaped oligomers. In agreement with this hypothesis, the predicted membrane-spanning region of the recently crystallized monalysin also begins at the top of the PFM and its deletion abolishes pore formation^17^.

The PFMs in aerolysin, epsilon toxin and parasporin-2 also contain N- or C-terminal peptides (CTPs), which have to be proteolytically removed for activation. These pro-peptides have been implicated in chaperoning the secreted protoxins and controlling oligomerization^5^,^31^,^32^,^33^ and their removal is most likely to occur subsequent to receptor binding and on monomer–monomer interactions^32^,^34^. The most striking feature of the pre-insertion strands is that they interact directly with the CTPs of aerolysin and epsilon toxin. In both cases, the CTPs create a bridge between the pre-insertion strands and the main body of the toxin by participating in β-sheets with both domains (Fig. 4, CTPs coloured in red). Thus, it is evident that removal of the CTP in aerolysin and epsilon toxin will reduce the energy required for the pre-insertion strands to disengage from the main body of the toxin and rearrange into the long β-hairpin. In the case of aerolysin, one of the pre-insertion strands is predicted to partially unfold in the absence of the CTP, possibly acting as a switch to initiate membrane insertion^32^, providing further evidence for the involvement of the pre-insertion strands in pore formation. Finally, removal of the CTP is likely to expose surfaces in the cap domains of these proteins required for complete interaction between the subunits and to promote oligomerization prior to pore formation^31^.

The mode of lysenin membrane insertion identified in this study is similar to that of the α-haemolysin family of small-βPFTs. The staphylococcal haemolysins and clostridial NetB toxin undergo a simple extension of a pre-stem region, which is tucked against the β-sandwich domain, to form the β-barrel pores without any significant changes to the remainder of the structure^23^,^25^,^26^,^35^. This mechanism is remarkably well-conserved within this family even in cases where the symmetry, subunit stochiometry and receptor-binding regions vary^27^. In a similar way, the lysenin pre-insertion strands unfold away from the PFM and rearrange into a long β-barrel. However, in contrast to α-haemolysin the PFM of lysenin undergoes a conformational change resulting in a collapse of ∼20 Å and an inwards compaction (Supplementary Movie 1). The pore model for aerolysin proposed by Degiacomi and co-workers^31^ derived from an 18 Å cryo-EM map combined with molecular dynamics of mutants locked in a pre-pore and quasi-pore conformations also bears similarities to the lysenin pore structure in that the overall domain arrangement of the receptor-binding domain 2 and the PFM (domains 3 and 4) are positioned similar to lysenin. The extension of the aerolysin β-hairpins, however, may begin, as in the case of lysenin, at the top of the PFM and include the pre-insertion strands. As more pore structures of the aerolysin family are solved to high resolution, it will become clearer how these toxins vary and to what extent the insertion mechanisms are conserved.

**Figure 5:**
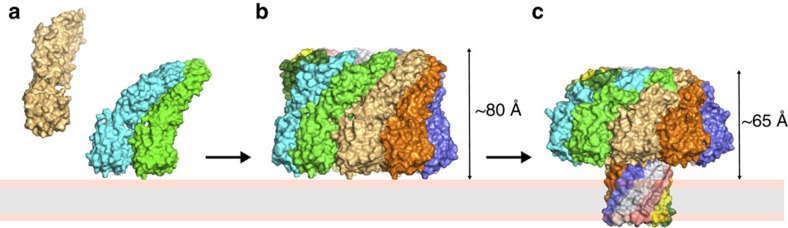
Model for the mode of action by lysenin. (**a**) Lysenin monomers (PDB ID 3ZXD) will initially bind to the target cell membrane through interactions between the receptor-binding domain and POC head groups. (**b**) On membrane binding, the local concentration increase of monomers will promote oligomerization, leading to a pre-pore assembly. Depending on the orientation of the monomers, the height of the pre-pore may be as high as 100 Å (the length of a lysenin monomer). (**c**) The membrane-inserted form of lysenin solved in this study will extend ∼65 Å above the membrane surface. The hypothetical pre-pore shown here was assembled by imposing ninefold symmetry to the monomeric crystal structure aligned in reference to the receptor-binding domain of the membrane-inserted form.

Based on our findings and previous work in the field, a model for pore formation by lysenin is presented in Fig. 5. The initial binding of soluble monomers to the membrane will be mediated through the receptor-binding domain to POC head groups (Fig. 5a) (ref. 9 and this study). This will additionally help to orient the toxin prior to pre-pore formation. Recognition of SM may also occur at this point^9^. This will be followed by oligomerization to a pre-pore state (Fig. 5b), as identified by AFM^10^,^36^ and electron crystallography^9^, through monomer–monomer interactions that may trigger the unfolding of the β-hairpins to a lower energy state and permit closer packing of the monomers. Oligomerization, a step that is obligatory prior to pore formation for all β-PFTs characterized to date, ensures that a sufficient number of β-hairpins are present for channel formation^9^ and satisfies the β-strand hydrogen-bonding potential in a hydrophobic environment such as the lipid bilayer^37^. Finally, β-barrel formation will create a pore in the bilayer (Fig. 5c), leading to the disruption of membrane homeostasis and ultimately to cell lysis.

## Methods

### Recombinant protein production and purification

For expression of lysenin, recombinant plasmid pHis-Parallel1-Lys was transformed into *E. coli* Rosetta 2 (DE3) cells (Merck, Darmstadt, Germany) and expression of lysenin was induced using the autoinduction system^38^ as follows. Cells (2 l) were grown in ZYM-5052 autoinducing medium supplemented with 100 μg ml^−1^ ampicillin and 34 μg ml^−1^ chloramphenicol and cultured at 37 °C for 3 h at 300 r.p.m., then for a further 24 h at 20 °C, 300 r.p.m. Cells were harvested by centrifugation and the cell pellet was lysed by 200 ml BugBuster Protein Extraction Reagent (Merck) containing 200 μl rlysozyme (1 kU μl^−1^) (Merck) and 200 μl Benzonase Nuclease (25 U μl^−1^) (Merck). The cell suspension was incubated on a rotating mixer for 25 min at room temperature and centrifuged at 16,000*g* for 20 min at 4 °C to separate soluble and insoluble fractions. The supernatant was loaded onto His GraviTrap columns (GE Healthcare Life Sciences, Little Chalfont, UK) following the manufacturer's guidelines. In brief, His-tagged proteins were bound to the affinity column using a buffer composed of 20 mM sodium phosphate, 500 mM NaCl and 20 mM imidazole, pH 7.4. The column was washed with a buffer composed of 20 mM sodium phosphate, 500 mM NaCl and 60 mM imidazole, pH 7.4. Recombinant toxin was eluted in a buffer composed of 20 mM sodium phosphate, 500 mM NaCl and 500 mM imidazole, pH 7.4. All purification steps were carried out at 4 °C. For buffer exchange and sample clean up, toxin containing eluate was applied to a PD-10 Desalting Column (GE Healthcare Life Sciences) and eluted in DPBS buffer pH 7.0–7.2 (Invitrogen). Protein concentrations were determined using the BCA assay (Fisher Scientific UK Ltd, Loughborough, UK).

### Haemolysis assay

Fresh whole blood from healthy individuals was collected by venepuncture from the University of Exeter Medical School, NIHR Exeter Clinical Research Facility, Diabetes and Vascular Medicine Centre into neutral tubes and 1 ml whole blood was immediately transferred into 20 ml DPBS buffer pH 7.0–7.2 supplemented with 1 mg ml^−1^ bovine serum albumin (BSA). Cells were washed three times with DPBS supplemented with 1 mg ml^−1^ BSA and resuspended to 3 × 10^7^ cells per ml in DPBS. Washed cells (3 × 10^6^ cells per well) were incubated with a twofold dilution series of purified recombinant lysenin (ranging from 500 to 0.015 ng ml^−1^) in DPBS in round-bottomed 96-well plates in a final volume of 200 μl. DPBS and 0.9% Triton X-100 were used as controls. Following incubation at 37 °C for 30 min and for 30 min on ice, intact cells were removed by centrifugation at 1,500*g* for 3 min at 4 °C and the supernatants (100 μl) were transferred to a flat-bottom plate to measure haemoglobin release by absorbance at 415 nm using a Model 680 Microplate Reader (Bio-Rad Laboratories Ltd., Hemel Hempstead, UK). The absorbance values for each sample were normalized by subtracting the absorbance value obtained for untreated cells and haemolytic activity (%) was calculated. The toxin dose required to lyse 50% of the cells (CT_50_) was determined by nonlinear regression analysis, fitting a variable slope log (dose) versus response curve, constraining *F* to a value of 50 (logCT_50_=logCTF−(1/HillSlope) × log(*F*/(100−*F*)). Results are presented as the mean of triplicate assays±s.e.m.

### Lysenin pore assembly

Lipid vesicles containing DOPC, porcine brain sphingomyelin and cholesterol at a molar ratio of 1:1:1 were prepared as follows. Lipids (Avanti Polar Lipids Inc.) dissolved in ethanol were mixed at the appropriate ratio and dried under a nitrogen stream. To ensure no residual solvent remained, lipids were further dried in a vacuum desiccator for 1 h. Lipids were hydrated by addition of buffer (50 mM Tris pH 7.4, 150 mM NaCl) and frozen in liquid nitrogen. Lipid suspensions were then thawed at 37 °C and the freeze–thaw process was repeated two more times. The lipid suspensions were then extruded 21 times through a 200 nm diameter filter using an Avanti lipid extruder and the resulting vesicles were extruded again through a 100 nm filter 21 times. Liposomes were then frozen at −80 °C until further use. Lysenin monomer (480 μl of 1.3 mg ml^−1^) was incubated with liposomes (34 μl of 20 mM lipid) at 37 °C for 30 min to allow binding and pore formation. Unbound lysenin was removed by ultracentrifugation at 50,000*g* for 45 min at 7 °C using a Beckman TLA 100 rotor. To screen for a suitable detergent that could efficiently extract and maintain the lysenin pores soluble, the pellets were resuspended in buffers containing 50 mM Tris pH 7.4, 150 mM NaCl and detergent (either 2.5% (w/v) β-OG or 30 mM LDAO or 40 mM C10E6 or 2% (w/v) DDM) and incubated at room temperature for 1 h with occasional shaking. Unsolubilized material was removed by ultracentrifugation as described above. For DDM extracted samples, solubilized lysenin oligomers were then bound to the Ni-NTA resin, which was subsequently washed with 20 vol of 50 mM Tris pH 7.4, 150 mM NaCl, 0.02% (w/v) DDM, followed by elution in 2 vol of 50 mM Tris pH 7.4, 150 mM NaCl, 500 mM imidazole and 0.02% (w/v) DDM.

### Specimen preparation and data collection

Specimens were plunge-frozen using a custom fabricated plunger at 4 °C. Lysenin oligomers (3 μl of ∼0.2 mg ml^−1^) were applied to copper 300 square mesh Quantifoil R1.2/1.3 holey-carbon grids (Quantifoil Micro Tools, GmbH) overlaid with graphene oxide (see below) and left to adhere for 30 s. The grids were then blotted from the specimen side for 10 s before being plunge-frozen in liquid ethane. Specimens were imaged on an FEI Titan Krios transmission electron microscope operating at an accelerating voltage of 300 kV. Micrographs were recorded in super-resolution counting mode using a Gatan K2 Summit direct electron detector at the end of a Gatan Quantum energy filter in zero-loss mode and an energy selecting slit width of 20 eV. The total dose on the specimen was 47 e^−^ per Å^2^ fractionated over 20 frames with a calibrated pixel size of 0.715 Å for the super-resolution micrographs.

### Image processing

Micrograph frame stacks were binned by two, subjected to drift-correction using MOTIONCORR^39^ and contrast transfer function (CTF) parameters were determined using GCTF^40^. An initial model for refinement was generated using EMAN2 (ref. 41). All subsequent image processing steps were performed with RELION^42^. Semi-automated particle picking was performed using the documented procedures^43^, resulting in 53,779 particles from 268 micrographs. Two rounds of 2D classification were used to remove incorrectly selected particles, particularly ones at the graphene layer interfaces. The remaining 42,830 particles were then used for initial refinement and reconstruction that led to a map with a resolution of 3.4 Å with C9 symmetry or 4.2 Å with no symmetry imposed. Movie particle extraction followed by per particle beam-induced motion correction and radiation-damage weighting^22^ resulted in a polished data set that was then subjected to 3D classification. Two of the 3 resulting 3D classes were combined and the resulting 29,329 particles were used for further refinement. The final map from these particles was sharpened with a *B*-factor of −61 Å^2^ and the ‘gold standard'^42^ resolution was 3.14 Å at a Fourier shell correlation (FSC) of 0.143 (Supplementary Fig. 4a). The accuracy of rotation and translation during refinement was 0.86 pixels and 0.33 pixels, respectively. Local resolution estimation was performed using RESMAP^44^ as implemented in RELION.

### Model building and refinement

The wild-type lysenin crystal structure^9^ (PDB ID 3ZXD) was used as a template for *de novo* modelling of the N terminus after the C-terminal 150 residues, which form the β-trefoil receptor-binding domain. All model building was performed in Coot^45^. The model is lacking the 9N-terminal residues, for which we could not see density. Refinement of the model to improve fitting, geometry and atom clashes was carried out using REFMAC 5.8 (ref. 46) with non-crystallographic symmetry constraints to account for the ninefold symmetry and secondary structure restraints generated by PROSMART^47^. Cross-validation of the refinement parameters used to avoid over-fitting was carried out by refining the model against the first unfiltered half map and comparing the FSC of the same model versus both half maps (Supplementary Fig. 4b).

### Graphene oxide specimen support preparation

Graphene oxide dispersion in H_2_O (Sigma) was diluted to 0.2 mg ml^−1^ in H_2_O and spun at 300*g* for 30 s to remove large aggregates. Quantifoil R1.2/1.3 holey grids were glow discharged for 1 min and 3 μl of the graphene suspension was added to the grids for 1 min. Grids were subsequently blotted briefly using Whatman No1 filter paper and washed three times on 20 μl drops of H_2_O (twice on the graphene side and once on the reverse side). Grids were then used for plunge-freezing without further treatment.

## Additional information

**Accession codes**: The cryo-EM maps of the lysenin pore have been deposited to the Electron microscopy Data Bank under accession number EMD-8015 and the refined atomic coordinates have been deposited to the Protein Data Bank under accession number 5GAQ.

**How to cite this article**: Bokori-Brown, M. *et al*. Cryo-EM structure of lysenin pore elucidates membrane insertion by an aerolysin family protein. *Nat. Commun.* 7:11293 doi: 10.1038/ncomms11293 (2016).

## Supplementary Material

Supplementary Figures Supplementary Figures 1-6

Supplementary Movie 1 The movie shows the transition of the water-soluble monomers to the membrane-inserted state as seen from the side and top (extracellular) views. The monomers (PDB ID 3ZXD) have been arranged in a hypothetical pre-pore assembly by imposing 9-fold symmetry on the monomer, oriented in respect to the receptor-binding domain of the membrane-inserted form. The colouring scheme is as in the main text: β-hairpin domain (olive), cap domain (pink) and receptor binding domain (cyan).
