# Supplementary material for: Cryo-EM structure of lysenin pore elucidates membrane insertion by an aerolysin family protein
Source: Nat Commun. 2016 Apr 6;7:11293. doi: 10.1038/ncomms11293 (PMC4823867; doi:10.1038/ncomms11293)
Supplement: Supplementary Figures — 1-6 [file ncomms11293-s1.pdf]

Supplementary Figures

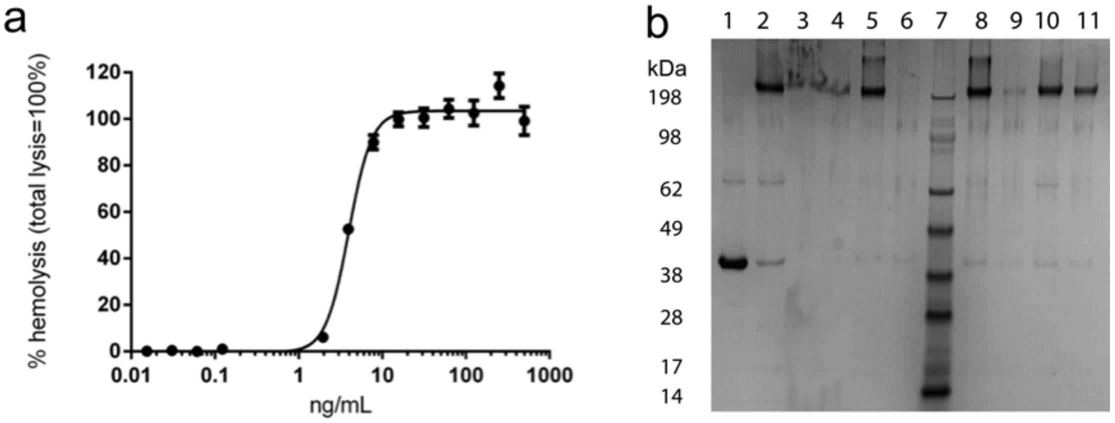

Supplementary Figure 1. **Lysenin activity and detergent optimisation**. **(a)** Human red blood cells (3 x 10^7 cells/mL) were incubated with a two-fold dilution series of lysenin for 30 min at 37 °C followed by measurement of hemolysis as described in Methods. **(b)** Detergent extraction and solubilisation of the lysenin pores. Following detergent extraction, samples were centrifuged and the samples prior to centrifugation (total) and resulting supernatants were analysed by SDS-PAGE. Lane contents: 1. Lysenin, 2. Lysenin plus liposomes, 3-4. LDAO total and supernatant, 5-6. β-OG total and supernatant, 7. Mw Marker, 8-9. C10E6 total and supernatant, 10-11. DDM total and supernatant.

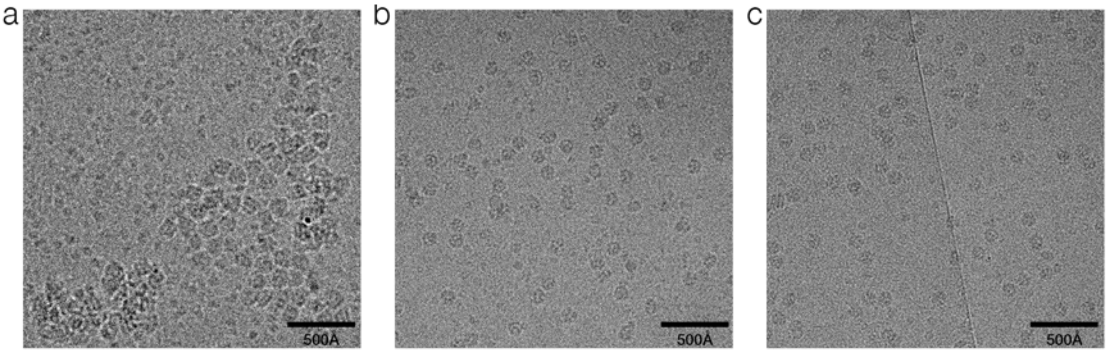

Supplementary Figure 2. Electron micrographs of the Lysenin pore. **(a)** Concentrated sample of the lysenin pore (~1 mg/mL) in pure ice with detergent micelles in the background. **(b, c)** Lysenin pore on graphene-oxide. **(c)** About 10% of the micrographs exhibit impurities in the graphene-oxide support. In this case it is a fold in a graphene-oxide flake. Particles in this area were removed during 2D classification.

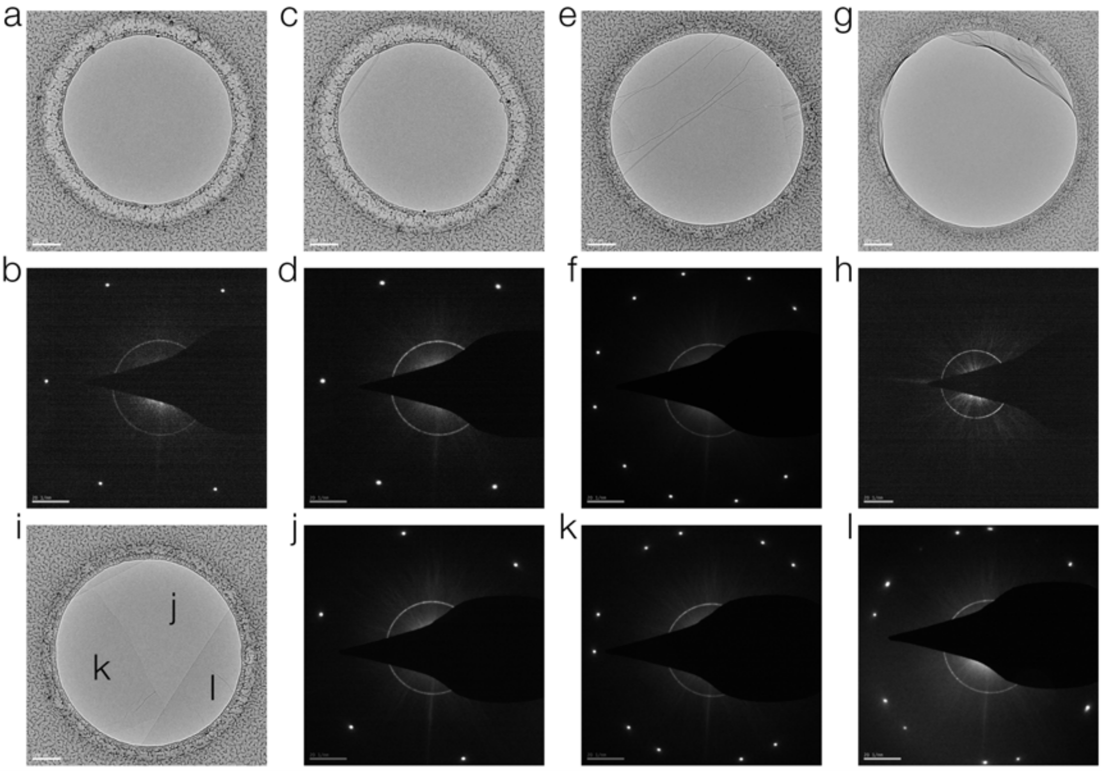

Supplementary Figure 3. Quantifoil grids with graphene-oxide support. (**a**) Perfect deposition of a graphene-oxide flake on a foil hole. The result is a near invisible support layer. (**b**) Diffraction image from the center of **(a)**. It shows 5 of the 6 characteristic diffraction spots for graphene, which confirms the deposition of only one flake. (**c**) Small fold inside a graphene oxide support layer. (**d**) Diffraction image from the center of **(c)**. (**e**) Multiple graphene-oxide flakes on one foil hole. (**f**) Diffraction image from the center of **(e)** showing twice the diffraction spots as in **(b)** and **(d)**, indicating two overlapping layers in the center of **(e)**. It might also be a result of a disturbance in the lattice through the visible fold. (**g**) Foil hole only slightly covered by graphene-oxide. (**h**) Diffraction image from the center of **(g)**, confirming that no support layer is present. (**i**) Sample foil hole with multiple flakes of graphene oxide in different regions of the hole. (**j, k, l**) Diffraction image of the indicated areas of **(i)**.

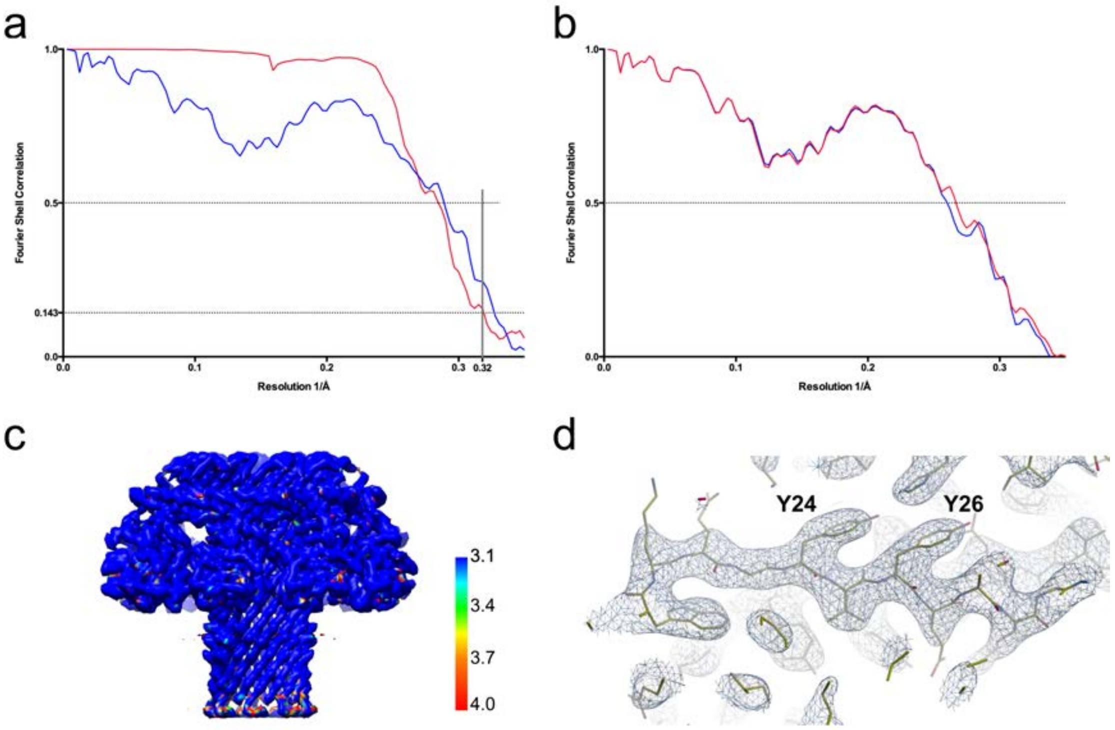

Supplementary Figure 4. Resolution calculations of the lysenin cryo-EM map. (a) Gold standard FSC of the two half maps (red curve) indicating a resolution of 3.14 Å at an FSC of 0.143. FSC of the refined model and the final map (blue curve) indicating an FSC at 0.5 at 3.45 Å. **(b)** Cross validation of the refinement parameters. FSC of model and the first half map, which was used for model refinement (red curve), and the FSC of the same refined model with the second half map (blue curve). The two curves overlap up to high resolution, indicating that the refinement weighting used did not result in overfitting. **(c)** Estimation of local resolution of the lysenin cryo-EM map using RESMAP. The colour scale from blue to red indicates the resolution range from 3.1Å to 4Å. **(d)** Example area of the cryo-EM density highlighting Tyr24 and Tyr26, which were involved in SM binding in the water-soluble form of lysenin.

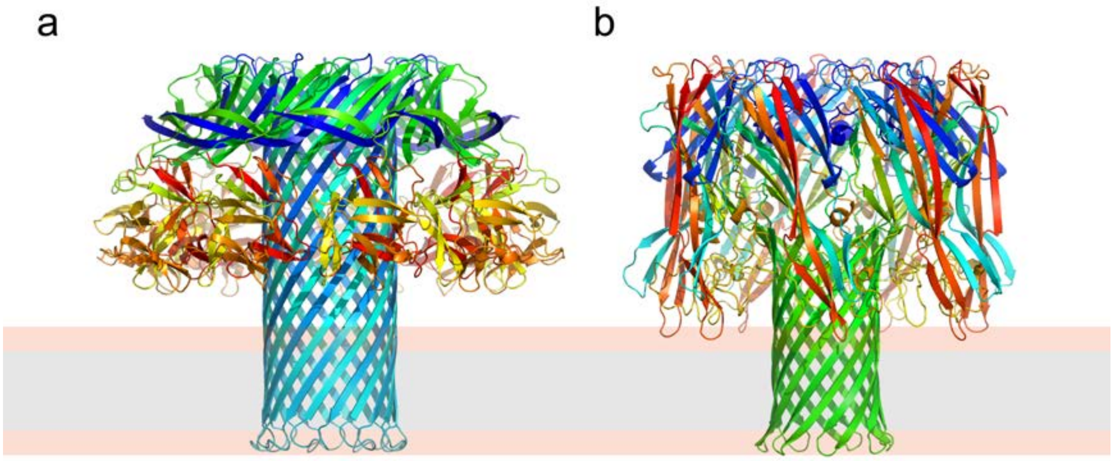

Supplementary Figure 5. Comparison of the membrane-inserted form of lysenin with staphylococcal α-hemolysin. The β-barrel pore of lysenin (**a**) spans the length of the assembly as compared to α-hemolysin (**b**) (PDB 7AHL). The approximate position of the bilayer is drawn with the phospholipid head groups shown in orange and the hydrophobic core in grey.

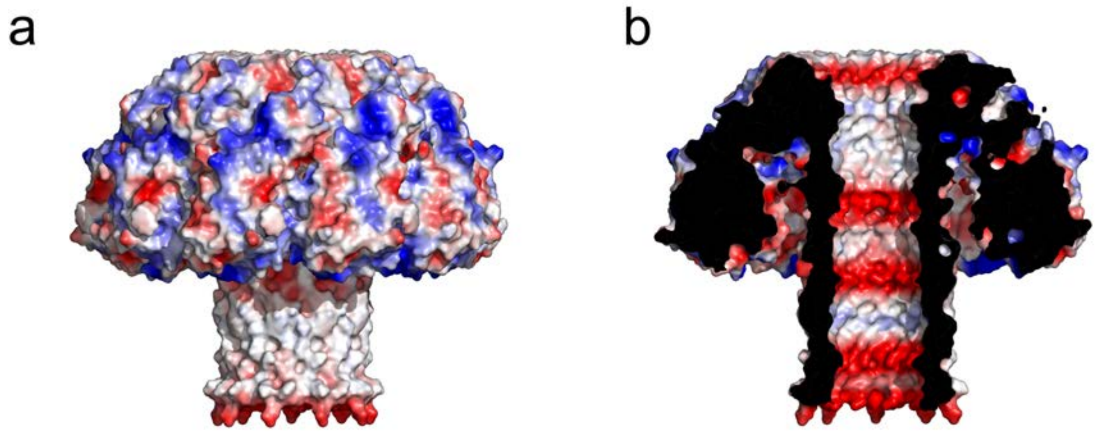

Supplementary Figure 6. Electrostatic potential map of the lysenin pore. (**a**) The exterior surface of the lysenin pore indicating the hydrophobic transmembrane region of the pore (neutral: grey) and (**b**), a sliced view showing the lumen charge distribution, which reveals 4 zones of negative charge (red). Positive charges are shown in blue.
